# Protocol: a systematic review+ (SR+) to combine associative and mechanistic evidence on the efficacy of face masks in reducing transmission of respiratory diseases

**DOI:** 10.1186/s13643-025-02973-2

**Published:** 2025-11-17

**Authors:** Trisha Greenhalgh, Rebecca Helm, Luana Poliseli, Sahanika Ratnayake, Alexandra Trofimov, Jon Williamson

**Affiliations:** 1 Nuffield Department of Primary Care Health Sciences, University of Oxford, Woodstock Rd, Oxford, OX2 6GG UK; 2 Department of Law, University of Exeter, Exeter, UK; 3 Department of Philosophy, University of Manchester, Manchester, UK

**Keywords:** Systematic review, Mechanism-informed systematic review, Systematic review + (SR+), Narrative review, Evidential Pluralism, Epistemic justice, Hierarchy of evidence, Risk of bias, GRADE, EBM+

## Abstract

**Background:**

Mechanistic evidence is evidence about how an intervention works. A 2023 Cochrane review, which was restricted to randomised controlled trials (RCTs), concluded that evidence on the efficacy of face masks was weak, conflicting and non-definitive. A 2024 narrative review, which included RCTs plus mechanistic evidence on masks and mask mandates, concluded that evidence of efficacy was strong, consistent and definitive. These strikingly contrasting conclusions reflect differences in how evidence is valued. Orthodox synthesis methods (e.g. those used for Cochrane reviews, informed by GRADE criteria) classify mechanistic evidence as lower quality than RCT evidence, but this position has been challenged by (among others) philosophers, non-RCT researchers and advocacy groups. We seek to include mechanistic evidence in a systematic review of mask efficacy.

**Method:**

Three overlapping work packages (methodology, review, philosophical analysis) will run concurrently. We will extend and refine the philosophical approach of Evidential Pluralism, which has been applied in a technique known as EBM+, to develop Systematic Review+ (SR+). SR+ will use Bayesian methods to support judgements of whether and to what extent interventions are effective. We will apply SR+ to face mask (and mask mandate) efficacy studies purposively selected for their epistemic contribution (the most robust and influential studies in each evidential category). We will consider whether SR+ adequately addresses philosophical objections to orthodox systematic review, including epistemological (does it adequately incorporate mechanistic evidence into reviews of efficacy?) and ethical (does it adequately address epistemic injustice, in which someone is wronged in their capacity as knower?).

**Discussion:**

We hope to produce a robust synthesis of evidence on face masks that will inform policy and a general methodology for incorporating mechanistic evidence into systematic reviews. We also hope to contribute to the scholarly literature on the philosophy of causality. Causal claims generally require at least two kinds of evidence: associative (to show that a change in one phenomenon is associated with a change in another) and mechanistic (to be confident that observed associations are causal). We hypothesise that orthodox systematic review, enhanced with mechanistic evidence, will be able to support stronger and more nuanced causal claims.

**Systematic review registration:**

INPLASY202550024, INPLASY202540045.

**Supplementary Information:**

The online version contains supplementary material available at 10.1186/s13643-025-02973-2.

## Background

### Evidence synthesis and the face mask controversy

The COVID-19 pandemic raised many questions about scientific evidence and how such evidence should be ranked and synthesised. Urgent policy questions about interventions at the height of the crisis (e.g. When should lockdowns be imposed and how long should they continue? Who, if anyone, should be required to wear masks and in what circumstances?) rekindled a long-running paradigm war. On one side was an ‘orthodox’ evidence-based medicine (EBM) school which, on the basis of an agreed hierarchy of evidence, focused almost exclusively on randomised controlled trials (RCTs), in which study participants are randomly allocated to receiving an experimental or control intervention [1, 2]. EBM’s hierarchy placed meta-analyses of RCTs above single RCTs, which sat above observational studies such as cohort studies and natural experiments, which sat above mechanistic evidence, which sat above (or sometimes even below) ‘anecdote’ and ‘opinion’.

On the other side was a ‘heterodox’, more pluralist, school which sought to embrace multiple kinds and sources of evidence, including but not limited to RCTs, and to evaluate each source of evidence on its merits [3, 4]. In particular, pluralists sought to combine effect size estimates from meta-analyses of RCTs (a form of associative evidence) with information on how that effect might have been achieved, or why a hypothesised effect was not achieved (a form of mechanistic evidence).

While the conflicts between these schools were not new, the pandemic provided stark illustration of how perspectives on scientific evidence were deeply held and fiercely defended, and how human lives and liberties depended on what kinds of evidence—and, especially, what methods of evaluating, ranking and synthesising evidence—were seen by policymakers, and the scientists who advised them, as the most credible. Disputes about such issues typically play out as clashes of methodology, but they can be traced back to more fundamental philosophical differences—ontological (the nature of reality), epistemological (ways of knowing) and axiological (what is of value). Some scholars have framed the ‘hierarchy’ approach to evidence as a form of epistemic injustice [5–7], defined as where someone is wronged because the knowledge they offer is dismissed as ‘low quality’ or they are unable to express it in language that meets the expectations of dominant voices [8].

In relation to face mask efficacy, an orthodox systematic review, analysing only RCTs and published in the Cochrane Library, concluded that the existing evidence was of variable quality and non-definitive [9]. The abstract reads thus:

*‘The low to moderate certainty of evidence means our confidence in the effect estimate is limited, and that the true effect may be different from the observed estimate of the effect. The pooled results of RCTs did not show a clear reduction in respiratory viral infection with the use of medical/surgical masks. There were no clear differences between the use of medical/surgical masks compared with N95/P2 respirators in healthcare workers when used in routine care to reduce respiratory viral infection.’ (page 1)* [9]

Given the sample of studies reviewed, these conclusions were appropriately cautious, but they were interpreted by the press and many members of the public (and, indeed, by one of the Cochrane review’s own authors) to mean that the review had shown that ‘masks don’t work’. This prompted Cochrane’s editor-in-chief to issue a clarifying statement [10]. Leaving aside the awkwardness of this incident for Cochrane’s reputation and internal relations, the question arises: what kind of *additional* evidence might have helped resolve the controversy?

As Table 1 shows, RCTs on masks are extremely difficult to conduct. While many have been published (see this review for a summary [11]), and almost all had what orthodox reviewers would call a high risk of bias. The question of whether it would be possible to design a RCT that adequately overcomes these biases is contested (see the ‘Discussion’ section). An alternative (or complementary) way of resolving the controversy on mask efficacy is to supplement the body of sparse, flawed, incomplete and contested RCT evidence with *mechanistic evidence*, an approach discussed in the next section.

**Table 1:** Limitations of the RCT design for testing the efficacy of face masks

Criterion	Quality standard
Prior assumptions	The RCT design assumes that outcomes are independent of one another. When this is not the case (e.g. in healthcare settings where prevalence of the disease is high), cluster randomisation (e.g. by hospital) should be used. Most RCTs of masks were not cluster randomised (often for resource reasons, since cluster RCTs need to be larger and involve more participants)
Study design	To assess mask efficacy, two things should be measured: (a) the wearer’s chance of becoming infected and (b) source control (i.e. the public health question of how much *other people* are protected if someone wears a mask). Most RCTs of masks looked only at the former, hence systematically underestimating efficacy. Studying source control is difficult, since outcomes generally need to be measured in large numbers of people who did not receive an intervention and may not have consented to participation in a study
Intervention development and piloting	A complex intervention should be optimised and piloted before testing in a definitive RCT. In relation to masks, attention needs to be paid to (among other things) filtration, breathability, comfort and fit. How and when the mask is worn should reflect the science of pathogen transmission. Few mask trials included an optimised intervention
Sample size and power	RCTs should be adequately powered. The sample size for an RCT of an intervention to prevent communicable disease transmission will vary with the disease prevalence. In reality, many mask trials were based on sample size calculations which assumed a significantly higher prevalence of the disease than actually occurred (i.e. they were underpowered)
Ethical considerations	Researchers must be in equipoise (i.e. have no good reason to believe that one arm of the trial will fare better than the other). For some groups (e.g. the clinically extremely vulnerable), many scholars consider that equipoise about the value of masking is unjustified and hence that it would be unethical to conduct a RCT
Setting	Studies conducted in one setting, even when internally valid, may have poor external validity (i.e. transferability to other settings). Lack of efficacy of advice to mask in a low-risk context (e.g. flu outbreak on a university campus) does not necessarily transfer to a high-risk context (e.g. a spreading pandemic, in which advice is more likely to be heeded)
Concealment of allocation	RCTs should ideally be ‘double blind’, i.e. neither participants nor assessors should know the allocation arm. It is not possible to conceal from someone whether they are wearing a mask (or advised to mask). ‘Sham’ masks (which give the appearance of protection but lack actual filtration efficacy) do not exist (but if they did, would likely be spotted as such)
Compliance	It is generally assumed that RCT results should be analysed on an intention-to-treat (rather than per-protocol) basis. Most mask trials did analyse on intention-to-treat, but some studies in which compliance was poor failed to distinguish ‘advice to mask’ from ‘actually wearing a mask’, leading to the flawed conclusion that masks don’t work
Outcome measures	Infection should be confirmed with a sensitive and specific test. In reality, mask trials used a range of outcome measures (variously, patient self-reports, clinical assessment, near-patient tests and laboratory tests)
Follow-up	Follow-up should be long enough and complete enough to identify an effect if it exists. In reality, many RCTs of masks had very short follow-up periods
Contamination and confounding	An RCT should compare masks with no masks, with no other differences between the arms. In reality, some RCTs included additional measures (e.g. handwashing) in the intervention but not the control arm Healthcare workers who mask at work may be infected at home, especially when community prevalence is high. Most trials of masking in healthcare workers failed to acknowledge the possibility that community transmission could have masked real differences in efficacy between respirators and medical masks
Combining studies	When undertaking meta-analyses, similar studies should be aggregated but dissimilar studies should not be. In reality, many meta-analyses of masks have inappropriately combined dissimilar studies
Assessment of adverse effects and harms	All potentially severe harms should be measured. RCTs that were powered to demonstrate or exclude a clinically significant effect size will be underpowered to detect rare harms

### Associative evidence, mechanistic evidence and Evidential Pluralism

Associative (sometimes called ‘probabilistic’ or ‘difference-making’) evidence is defined as evidence that two phenomena are linked such that differences in one are accompanied by differences in the other [12]. Among association studies, RCTs are particularly prized because they can increase confidence that an observed correlation is not spurious, i.e. that it is attributable to some mechanism by which A causes B. Mechanistic evidence is defined as evidence which helps to shed light on the factors and interactions that are responsible for (that is, which *cause*) a phenomenon [12, 13]. The mask example shows that mechanisms span multiple disciplines, from microscopic (e.g. how the size, shape and charge of a particle influence how well it is filtered by different kinds of mask material) to societal (e.g. how public opinion can be influenced by mass media). Examples of mechanistic studies in relation to masks are shown in Table 2.

**Table 2:** Summary of types of mechanistic study relevant to the mask causality question

Topic	Study type
How particles (aerosols and droplets) spread	Laboratory studies (e.g. fluid dynamics)
Spread of infectious diseases in a community	Mathematical modelling, taking account of disease prevalence, contagiousness and the susceptibility of the population, as well as confounders such as concurrent interventions
Design of masks and mask materials	Material and engineering studies of filtration efficacy, breathability (resistance), fit and seal (a measure of leakage when worn) and potential for contamination. Design studies of how masks can be made to fit different shaped faces
Occupational hygiene	Studies of how employers might protect their staff from hazards
Human attitudes and behaviour (compliance with mask advice and mask mandates)	Qualitative (e.g. interview) and quantitative (e.g. questionnaire) studies of what people feel about masking. Anthropological, socio-material and sociological studies of the meaning of masking in different societies. Studies of how best to communicate information and advice about disease outbreaks and masking to different target audiences. Qualitative and quantitative studies of how (mis)information spreads on public social media platforms (e.g. Facebook) and among private groups (e.g. WhatsApp)
Development and implementation of policy	Legal and policy analyses (case studies of masking legislation and policies in different settings). Usually in the form of richly described and contextualised case examples
Economics of masking	Studies to estimate the costs and benefits of particular approaches to masking policies

The first three rows of Table 2 relate to the question ‘how effective are masks and other face coverings at blocking the ingress or egress of infectious particles in a respiratory outbreak?’, and the last four rows pertain to the question ‘how effective are face mask mandates at achieving uptake of masks or other face coverings in a population?’. The mechanistic evidence to answer the first question comes predominantly from the physical sciences (e.g. physics, engineering, chemistry). The evidence to answer the second question comes predominantly from the social sciences (e.g. psychology, sociology, anthropology, law, economics) and public health. Both questions must be answered to explore the full chain of causation in relation to mask efficacy. Given that some but not all published RCTs of masks have produced null results, the main role of mechanistic evidence in this example would be to explain why masks and mask mandates might, *ceteris paribus*, be expected to work and identify testable hypotheses about why their efficacy might be limited in certain specific designs and contexts.

**Fig. 1:**
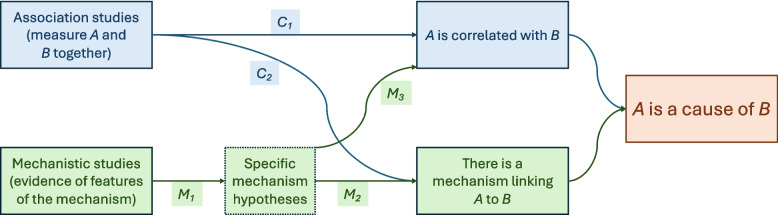
Evidential relationships posited by Evidential Pluralism. Adapted under Creative Commons licence from Shan and Williamson [4]. Comparative association studies (which measure the putative cause and effect, often together with potential confounders) yield direct estimates of the correlation between the two (C_1_), and, in certain cases, can also indirectly confirm that the measured correlation is attributable to some underlying mechanism, rather than bias or confounding, say (C_2_), but the presence of a suitable mechanism is more directly confirmed by hypothesising key features of the mechanism (M_2_) and testing for the presence of those features (M_1_). In certain cases, a well-established mechanism can also raise confidence that the putative cause and effect are genuinely correlated (M_3_)

Evidential Pluralism is a systematic approach to combining associative and mechanistic evidence developed by philosophers [13–16] and embraced by health researchers in an emerging tradition known as EBM+ [4, 13]. It is illustrated schematically in Fig. 1.

According to Evidential Pluralism, establishing that A is a cause of B requires establishing the existence of both an *association* (correlation) between A and B and a *mechanism* connecting the two. Mechanisms can be systematically explored by hypothesising key features (such as mediating variables, or key entities or activities of the mechanism) and performing studies (known as ‘mechanistic studies’) to test whether those features are present. Finding such features to be present and active confirms causation, while finding them to be absent or inactive disconfirms causation. Either way, mechanistic studies can be informative.

### Aims and research questions

The study has two aims which will be pursued in parallel. First, to produce a novel synthesis of the literature on face masks and mask mandates in reducing transmission of respiratory infections that incorporates mechanistic evidence in a more systematic way than previous (e.g. narrative) reviews. Second, to use this example to develop a new, philosophically informed, approach to evidence synthesis, known as systematic review + (SR+) which will help reviewers and policymakers assess the evidence base for an intervention. SR+ will be designed to integrate mechanistic evidence from across academic disciplines and other relevant sources with available associative evidence (notably, from RCTs of interventions).

Our research questions are:

1. How can an existing philosophical approach (Evidential Pluralism) be extended and refined to produce a widely applicable method (systematic review +, SR+), for integrating associative and mechanistic evidence in systematic reviews of interventions?
2. How can SR+ inform the specific controversy about the efficacy of face masks and mask mandates in reducing transmission of respiratory diseases? How does it compare with other theory-based review methods (e.g. narrative review, realist review) in this regard?
3. To what extent can SR+ overcome epistemological objections to orthodox systematic review (e.g. that it fails adequately to incorporate mechanistic evidence)?
4. To what extent can SR+ overcome ethical objections to orthodox systematic review (in particular, epistemic injustice)?
5. What are the strengths and limitations of SR+ in evidence synthesis? In what contexts can it add value to evidence synthesis? When and how should it be combined with other review methods?

## Method and analysis

### Management and governance

The study is funded by a UKRI programme for interdisciplinary research. The chief investigator is Jon Williamson (Philosophy); the work is co-led by Trisha Greenhalgh (Public Health) and Rebecca Helm (Law). An interdisciplinary advisory board provides further interdisciplinary expertise and scrutiny, including but not limited to realist review, orthodox systematic review, health policy, mathematical modelling, aerosol science and analytic philosophy.

### Study design

In-depth case study applying philosophical methods to examine published empirical research studies, of face masks and mask mandates. We will pursue three work packages concurrently: developing SR+ methods, conducting the mask review and analysing the adequacy of the approach from a philosophical perspective. We consider these in turn below.

### Work package 1: develop SR+ methods

The SR+ approach will be developed by extending and refining the existing Evidential Pluralism approach, whose development and application to medical topics have been outlined previously [4, 12, 16, 17]. To build on this work, we will take two approaches. First, we will develop ways of informing *qualitative* (i.e. non-numerical) judgements relating to *whether* an intervention is effective. Second, we will develop ways of *quantifying* such judgements. Among other approaches, we plan to use new Bayesian methods to estimate how confident we should be that an intervention is effective and produce a numerical estimate of effect size. The technique will use a probabilistic graphical model of the evidential relationships posited by Evidential Pluralism as a device to calculate confidence in effectiveness and determine which kinds of study would best fill remaining gaps in the evidence base. This methodological work will progress in parallel with the specific review described in the next work package, adapting as needed as data emerge.

### Work package 2: undertake a SR+ of face masks and mask mandates

We will begin with a published narrative review on masks and mask mandates for the control of respiratory infections led by one of us (TG), which identified multiple studies including RCTs, observational studies and various kinds of mechanistic evidence [18]. We will use (and, where appropriate, extend) the multi-disciplinary primary evidence collected for that review, both associative (RCTs and observational studies) and mechanistic (relating to mask materials, mask performance, and the social and behavioural aspects of mask wearing). Using the methodology of SR+ developed in work package 1, we will bring these streams of evidence together as described in detail below.

The first sub-task of work package 2 will be, from the hundreds of primary studies and existing systematic reviews (of which over 60 have been published since 2020), to identify and collate a workable sub-sample of studies which can be used as a ‘substrate’ for refining and testing philosophical concepts on associative (RCT and observational) evidence. Broadly speaking, and using the PRISMA methodology [19] adaptively to take account of our philosophical aim, we will seek a maximum-variety sample of each kind of evidence, taking account the likely epistemic contribution (i.e. prioritising studies that are theoretically coherent, well-designed, adequately powered and using interventions that have been optimised, and are highly regarded by scholars in their field). We will explicitly seek studies which addressed the needs of marginalised or vulnerable groups and examine the extent to which such groups were considered across all studies in the sample.

For example, from dozens of RCTs of face masks in respiratory outbreaks, we will select trials in occupational settings (e.g. hospitals—see for example [20]), community-based trials with different units of randomisation from households [21] to entire villages [22] and trials in specific settings such as the Hajj pilgrimage [23]. From around 200 observational studies (see the systematic review summarising many of these [24]), we will select examples of classical epidemiological (cohort and case–control) studies (see for example [25]), database-derived real-world evidence (for example [26]) and ecological studies and quasi-experiments related to policy change (for example [27]). Studies will be selected independently by two reviewers with topic knowledge; differences will be resolved by discussion among the wider team, with input from expert advisers as needed.

Expressed in the conventional ‘PICO’ terminology, the details of associative studies to be considered for this part of the review are:

- Participants or population: any setting where masks or respirators are tested.
- Intervention: mask (cloth, medical, surgical) or respirator.
- Comparator: for associative studies, either no mask/respirator or a different kind of mask/respirator.
- Outcome: whether, to what extent and why the introduction of mask wearing affects the incidence of respiratory infections. Whether existing studies—both associative and mechanistic—are adequate to answer these questions.

Information sources will comprise electronic databases (chiefly PubMed, Embase, Social Science Citation Index and Psyclit), sources known to the authors, topic experts in the field, with no date restrictions. Topic experts will be sourced from authors’ existing contacts and also from published papers; they will contribute both by identifying additional papers and by helping the research team interpret papers already identified. Eligibility criteria for inclusion are as follows: empirical research studies (including evidence syntheses as a source of such studies) on the association between masking and reduction of respiratory disease transmission and on specific mechanisms underlying this association. Peer reviewed literature will be prioritised but if there is insufficient evidence on important aspects of the review question, publicly available preprints will be considered. We will apply no restrictions to study design, country of origin or language. Studies published in languages not spoken by the review authors will be translated (or data extraction undertaken directly) by multilingual colleagues. We anticipate that a wide range of designs will be relevant, including a preponderance of laboratory, qualitative and occupational health studies for the mechanistic component. Details of the search strategy and data management plan for this work package are given in the Appendix. Authors of primary studies will be contacted for raw data or additional information as appropriate.

Data extraction will be undertaken by two authors independently using data extraction tools and key aspects of the study appropriate to the study’s design; the detail of these will be developed iteratively as the review unfolds. As noted above, the study seeks to draw upon, challenge and extend the GRADE criteria and methodology (including risk of bias tools) for ranking and evaluating empirical evidence. With that in mind, we will use formal evidence synthesis tools reflexively and critically rather than as a source of settled truth. Using appropriate tools and checklists, primary studies will be critically appraised for trustworthiness (internal validity), e.g. risk of bias. As the use of such tools involves subjective judgments and is dependent on the expertise of the reviewers, input of topic experts will be sought as appropriate (e.g. from clinicians, occupational health and safety, engineers, aerosol scientists, statisticians).

Data extraction and synthesis will proceed as follows: Tables will be prepared of key studies, including author/year, study design, methods, sample, findings, strengths/limitations and comments.

Where appropriate, formal meta-analysis techniques will be applied to quantitative data to gain an estimate of effect size and confidence interval. Pooling will be limited to data collected from substantially identical studies, or those where there is a clear mechanistic justification for considering the conditions to be functionally equivalent. Where studies are too heterogeneous to justify meta-analysis, disaggregated data will be presented and an attempt will be made to understand how differences in study protocol and conditions explain the differing outcomes.

Inconsistencies between study outcomes will be elucidated by exploring the cause of the inconsistency. Hypotheses about subgroup effects will be tested as appropriate if and when they emerge as the review unfolds. Sensitivity analyses will be undertaken as appropriate if and when they become necessary as the review unfolds.

The findings from the review of associative evidence will be used to inform the second sub-task in this work package, namely to apply philosophical concepts to assess a sample of mechanistic studies. We will systematically select high-quality examples of studies relating to the proposed mechanism of action. Examples of these mechanisms (which operate at different levels) are shown in Table 3 (efficacy of masks) and Table 4 (efficacy of mask mandates). Qualitative evidence will be analysed thematically and with attention to key theories (e.g. of motivation, social influence), and combined using the hermeneutic cycle in which each new data source is used to refine and enrich the understanding of the whole. In this way, rich explanations will be generated of *how* an effect may be obtained, should one exist, or why such an effect is not obtained.

**Table 3:** Candidate mechanisms which could contribute to causality arguments about the efficacy of masks and respirators

Category	Hypothesised mechanism(s) and examples
Spread of infectious diseases	How a disease spreads, taking account of prevalence, contagiousness and susceptibility [28]. How spread differs in highly contagious versus less contagious diseases and in situations where a proportion of the population is immune
Aerosol formation, transportation and survival	How small particles become aerosolised [29]; size distribution of different droplets produced during respiratory events (coughing, sneezing, speaking, breathing) [30]; how aerosolised particles are transported in air currents (e.g. fluid dynamics of cough plumes and effects of ambient air currents, ventilation systems and room geometry on aerosol dispersion and concentration) [31]; mechanisms affecting airborne pathogens’ viability (e.g. ambient conditions such as humidity, temperature and CO_2_ level) [32]
Generation of aerosols by host organisms when infected with a pathogen	Activities such as speaking or singing (including the intensity and duration of these activities) [33]. So-called aerosol-generating medical procedures (e.g. intubation, bronchoscopy) [34, 35]
Interaction between mask materials and suspended particles	Filtration (including impaction, interception and diffusion), affected by fibre size, packing density and layering, and the concept of most penetrating particle size (MPPS); deposition (due to gravity or inertia); adsorption (relating to surface interactions such as van der Waals forces and electrostatic attraction); absorption (by which liquids may be taken up into mask materials); electrostatic interferences (how electrically charged fibres can attract and capture charged particles, and how charge can degrade over time with humidity); how pore size impacts breathability and filtration efficiency [36–38]
Effect of mask materials on pathogens	Including killing (e.g. via antimicrobial coatings such as silver or copper ions); persistence (e.g. how the microenvironment within the mask may affect survival of trapped pathogens); and permanent capture and retention (e.g. if mask materials bind pathogens securely, they will remain in the mask and not become re-aerosolised) [39]
Respiratory airflow and aerosol deposition	How air flows within the respiratory tract (nasal passages, trachea, bronchi, bronchioles, alveoli) and how this structure influences airflow patterns and particle deposition at different locations (e.g. what size particles reach the alveoli) [40]. Particle deposition within the respiratory tract, including inertial impaction (larger particles are unable to follow airflow direction and impact airway walls, especially at bifurcations), gravitational sedimentation (smaller particles settle out of the airflow due to gravity, particularly in the smaller airways and alveoli where airflow is slow) and Brownian diffusion (very small particles exhibit random motion due to collisions with gas molecules, increasing their likelihood of contacting and depositing on airway surfaces, especially in the alveoli) [41, 42]. How breathing patterns (tidal volume, breathing rate) influence airflow and deposition (e.g. how deeper, slower breaths may lead to greater deposition in the lower respiratory tract) [43]. How mask leakage (e.g. due to poor fit) can alter airflow patterns, allowing unfiltered air (and aerosols) to bypass the mask and be inhaled directly into the respiratory tract [44, 45]
Mask performance under standardised conditions	Fit factor testing (e.g. using challenge aerosols and measuring concentrations inside and outside the mask); breathability (which affects user comfort and compliance); aerosol-blocking ability, which includes the particle size range against which different types of masks (e.g. surgical masks, N95/FFP2/KN95 respirators) are tested and their respective filtration efficiencies for those sizes. Nominal filtration efficiency (under ideal laboratory conditions) differs from real-world effectiveness, which is influenced by fit and user behaviour [46]
Influence of mask wearing on human physiology and psychology	How physiological biomarkers, including oxygen and carbon dioxide levels, are affected (or why they are rarely affected) by mask-wearing in both healthy individuals and specific vulnerable populations (e.g. those with severe pre-existing lung disease), or during activities (e.g. strenuous exercise), and how this varies with mask type [47, 48]. How mask-wearing may produce a perception of breathlessness (dyspnoea) or claustrophobia, e.g. via a sensation of warmth or humidity, affecting compliance [47]

**Table 4:** Candidate mechanisms which could contribute to causality arguments about the efficacy of mask mandates

Category	Hypothesised mechanism(s) and examples
Policy and legal mechanisms	Direct enforcement (e.g. fines or other penalties for non-compliance) and visibility of enforcement (e.g. signage, police presence) will increase compliance [49, 50] Timing (mandates can get community masking in place quickly) can ‘flatten the curve’ of exponential transmission [51] The wording and scope of a mandate, as well as the nature and stringency of enforcement, will influence uptake [52, 53] Mandates can be legally challenged on the grounds that they violate citizens’ and businesses’ constitutional rights [54] or people’s human rights generally [55]. or that the legislature lacks the power to issue the mandate [56, 57] Mandates provide a just and fair way of reducing transmission to disadvantaged and vulnerable people [58] Political and ideological views of local decision-makers can increase whether (and how quickly) mask mandates are implemented [59, 60]. National directives (e.g. banning mask mandates in schools) can over-ride local or regional mandate policies [61] Mask mandates for children have been opposed on human rights grounds (as potentially harmful and therefore a breach of a child’s best interests), since children are at low risk of serious complications and mainly seen as disease vectors to more vulnerable people (e.g. grandparents) [62] Top-down (mandated, national or regional) implementation mechanisms work best when they are synergistic with bottom-up (voluntary, individual and community-based) responses [63]
Mechanisms for institutional compliance	Health and safety requirements for businesses, schools, hospitals and other organisations can create a consistent environment where mask-wearing is expected and normalised among both employees and customers/clients [64–66] Organisations under such mandates can more easily refuse entry or service to those not complying than if the same policy lacked a mandate [64–66]
Communicative mechanisms	Mandates (to the extent that they provide a clear and consistent message) minimise ambiguity about whether and when mask-wearing is required, simplifying public health messaging and reducing confusion [67] Positive messaging and public health campaigns associated with mandates can reinforce the benefits of mask-wearing [68] Communicative messages can reinforce social responsibility and behaviours intended to protect the vulnerable [67] Public health messaging in rapidly unfolding public health crises must balance the precautionary principle with transparency (e.g. about the uncertainty of recommendations) and epistemic humility [69] A communicative message will be less effective if the intended recipient does not find the source credible, finds the message runs counter to their shared world view and values, contradicts what they believe to be true or conflicts with their cognitive style [70]
Social norms, conformity and civic responsibility	Mandates signal that mask-wearing is the expected behaviour, increasing social pressure to conform. People are more likely to comply when they perceive that others are doing so [71] The perception of a collective effort can increase compliance, as individuals feel they are contributing to a shared goal and the public good. Wearing a mask can be a symbol of social solidarity (‘we’re all in this together’) [67, 72]
Group identification and peer pressure	In communities with strong social cohesion, peer pressure can be a powerful motivator for compliance with mask mandates [73] Conversely, some group identification (e.g. with libertarians [74] or conspiracy theorists [75]) may help explain non-compliance with mask mandates [72]
Altruism	Mandates can reinforce the altruistic motives (a selfless concern for the well-being of others) held by some people. In such circumstances, people choose to wear a mask not primarily for their own protection but to reduce the risk of transmitting to others (especially those who are vulnerable) [76]
Beliefs	People who believe that SARS-CoV-2 infection can be serious, and that masks are effective in protecting against it, are more likely to comply with mandates and vice versa [77, 78]
Risk perception and fear	People who fear the consequences of SARS-CoV-2 infection are more likely to comply with mandates [67, 79] Mandates can heighten awareness of the severity of the pandemic, increasing perceived risk and motivating individuals to take protective measures. The official nature of a mandate reinforces the seriousness of the situation [80] People with higher risk perception are more likely to follow a mandate [80, 81] Masking may (it is hypothesised) give a sense that one is protected, thereby reducing compliance with other protective behaviours (‘risk compensation’) [82]
Authority and trust	People are more likely to comply with mandates issued by trusted authorities (e.g., public health officials, government leaders) [69, 72] Criminal and punitive approaches to enforcing mask mandates can undermine trust and reduce compliance [55] Where polarisation of views exists, bipartisan endorsement of mandates increases trust (and hence compliance) [72] The perception that the mandate is based on scientific evidence increases compliance [67]
Habit formation	Consistent enforcement of mandates can help establish mask-wearing as a routine behaviour, making it more likely to persist even after the mandate is lifted [83]
Media and public discourse	Mandates often generate media attention, which can increase public awareness of the importance of mask-wearing Misinformation in mainstream or social media may increase resistance to mask mandates [72]
Community level action	Mandates can spur community level action, such as volunteer groups distributing masks, or local businesses providing mask wearing encouragement [67]
Economic and practical mechanisms	Mandates may be accompanied by the distribution of free or low-cost masks, reducing the financial burden of compliance [84] Making masks readily available increases the ease of compliance. Mask mandates may be associated with an increase in the price of masks [85]

The third sub-task in work package 2 is to integrate the two streams of evidence (associative and mechanistic) using the qualitative and quantitative philosophical techniques developed in work package 1 above. An additional challenge to synthesising mechanistic evidence on a topic as complex as masks is that the various mechanistic influences shown in Tables 3 and 4 may not be independent of one another but interact in complex (i.e. non-linear) ways.

The fourth sub-task is to compare these findings to those obtained from other methods of evidence synthesis. Four approaches will be compared:

- The abovementioned SR+ on masks and mask mandates;
- Relevant sections of the review by Jefferson et al. on non-pharmaceutical interventions, which used Cochrane methodology (i.e. GRADE criteria) and explicitly excluded mechanistic evidence [9];
- A previous narrative review by Greenhalgh et al., which included mechanistic evidence but did not adopt a systematic approach to examining or combining such evidence [18]; and
- A theory-informed review of selected elements of mask policy, undertaken on the same sample of studies that we will use for the SR+. This will be undertaken using appropriate methodology (e.g. RAMESES methodology for realist [86] or meta-narrative reviews [87]), which will include examining policymakers’ theories of change.

Thus, we will test the same SR+ methodology on contrasting bodies of evidence: from the biological and physical sciences (mechanisms of respiratory disease transmission and mask efficacy) and from the social and behavioural sciences (mechanisms by which mask mandates may promote uptake of face masks). In addition, we will selectively use metanarrative review methods [87] to examine the over-arching paradigms within which mask research, and the methodology of evidence synthesis, have evolved. Metanarrative review draws on Kuhn’s notion of paradigms; it highlights that findings must be understood in terms of the ‘normal science’ (core assumptions, methods and prevailing debates) of each discipline.

In sum, this task will, in relation to the face mask example, assess the extent to which different approaches to incorporating mechanistic evidence (SR+, narrative review, realist review, metanarrative review) can cover the totality of evidence (mechanistic plus experimental/observational), whether from the social sciences, physical sciences or other sources, and overcome the limitations of orthodox approaches which deem such evidence out of scope.

### Work package 3: philosophical analysis

This work package will investigate, from a philosophical perspective and using the face masks example, how far SR+ mitigates objections that have been raised to orthodox systematic review. First, building on previous work [4, 12, 16], we will investigate the objection that orthodox systematic review is epistemologically deficient because it fails to review mechanistic studies which can contribute to a judgement on whether an intervention works. While SR+ clearly addresses this objection to some extent by including mechanistic studies, the unanswered question is whether it does so adequately and optimally. Our task here will be to ascertain whether SR+ is entirely immune from the epistemological objection: does it allow all evidence to be incorporated on its merits (including but not limited to study design, study quality and external validity)?, and to see how other methods, such as narrative review and realist review, compare.

Secondly, we will investigate an ethical objection, namely that orthodox systematic review perpetuates specific injustices, e.g. by systematically sidelining the knowledge of the patients, citizens and front-line staff affected by the interventions [5]. The task here is again to ascertain whether SR+ is entirely immune from this objection, and to see how alternative methods, such as narrative review and realist review, compare. Both these subtasks will be undertaken through philosophical analysis and interdisciplinary discussion. Subject to resources, other examples may be brought in to provide complementarity (e.g. of different kinds of epistemological or ethical challenges).

## Discussion

### RCTs, mechanisms and causality

This ambitious study is explicitly interdisciplinary, seeking to contribute not only to specific policy decisions about whether and in what circumstances masking should be recommended or even mandated, but also to wider scholarly debates about the place of non-RCT evidence, the methodology of evidence synthesis and the philosophical literature on the assessment of causality.

As every student of epidemiology knows, evidence of association does not, on its own, establish causation, though strong and consistent evidence of association can make a causal link probable [12]. Epidemiologists consider the RCT a methodological gold standard in this regard, arguing that provided the sample size is large enough, the RCT design will mitigate the biasing effects of known, unknown and poorly understood confounders. This makes it plausible that any differences in outcomes between the study arms (i.e. the experimental group and the control group) are *caused by* the intervention, assuming it is executed faithfully, though *how* this effect occurs may remain a mystery.

This line of reasoning can be criticised on several grounds. In many practical contexts, high-quality RCTs are impossible to conduct [3, 88]. In particular, because of resource constraints or limited numbers of available participants, or complexity of intervention, a trial may be underpowered (i.e. too small to test its central hypothesis), leading to an imprecise estimate of effect size which overlaps the line of zero effect. Allocation to study arms may not be genuinely random (some comparative studies, for example, allocate sequentially), and it may be impossible for that allocation to be blinded (e.g. a person will know whether they are wearing or not wearing a mask), leading to allocation bias and assessment bias respectively. The study sample (e.g. clinic patients) may be unrepresentative of the wider population of people with the condition (hence, findings will not be *directly* relevant to certain groups), particularly since participants will need to self-select into a study and particular factors may be associated with a desire to participate (or not). By chance, important baseline characteristics (e.g. age, gender, disease severity or poor prognostic markers) may be (and in practice likely often are) unevenly distributed among the trial arms. Compliance with a drug or non-pharmaceutical intervention (e.g. masking) may be low; some participants in the ‘control’ arm may receive the intervention (e.g. some ‘control’ participants may choose to wear a mask even if allocated to a ‘no mask’ arm); and some people who comply with masking in the intervention arm of a study (e.g. masking consistently while at work) may nevertheless be exposed in other unmasked situations (e.g. at home, when the incidence of the disease is high in the community). Clinically important outcomes may go unmeasured. The study duration may be too short. A variable proportion of participants may be lost, leading to follow-up bias. All these situations will lead to under-estimation of effect size. Trials that do not produce the desired result are less likely to be published (publication bias).

In addition, interventions tested in RCTs may not be optimised (partly because they fail to take account of mechanism), leading to a measured effect size that under-estimates the potential impact of the intervention. This limits the utility of the results and clouds the potential for a modified intervention to be more effective. Insufficient attention to mechanism, combined with limitations in the representativeness of populations enrolled in RCTs, means that it is difficult to make accurate predictions relating to which populations and which contexts the results will generalise to. Trials may also use narrow testing situations or highly selected samples of participants (not too old, not too ill, not too young, etc.), leading to overestimates of the likely benefits in real-world situations and lower actual impact when the intervention is transferred to the real world. For these reasons (among others), RCTs are rarely sufficient in practice to establish causality. In relation to mask efficacy, by the time a RCT has been funded and organised, the prevalence of the disease may have fallen, reducing the power of a trial to demonstrate an effect size if one existed.

Causality occurs at many levels from molecular to global, and over many timescales (from nanoseconds to aeons) [89]. Where interventions are aimed at humans who are expected to comply with them, the causal influences include the culturally shaped symbolic meaning which that intervention holds for individuals (e.g. whether masks are seen as ‘protections’ or ‘restrictions’) and also structural and practical considerations such as affordability and opportunity cost. To understand these chains of causation (and particularly, to unpack issues of acceptability and compliance), evidence of the lived experience of the condition and the proposed treatment is needed [90]. Many different forms of mechanistic evidence will therefore be needed to establish causality, and mechanistic evidence comes in many forms depending on the nature of the phenomena [91]. Broadly speaking, then, those who seek to use mechanistic evidence to generate causal explanations tend also to take a pluralist perspective (i.e. embracing multiple kinds of evidence). Causality may be linear and predictable (in which case, an intervention will have a stable effect size in comparable prevailing conditions) but it may be non-linear and not fully predictable (in a complex system, for example, we talk about *generative* causality which needs to be modelled dynamically) [92].

When examining causality, epidemiologists often cite criteria developed by the British statistician Sir Austin Bradford Hill, who designed and led the first ever RCT (to test the efficacy of streptomycin in patients with pulmonary tuberculosis) [93, 94]. While Bradford Hill was convinced that benefits demonstrated in RCTs were vastly more trustworthy than findings from studies which used historical controls or no controls at all, he also considered that an RCT finding on its own was not adequate evidence that the drug had *caused* the observed benefits. He stressed that one must also take into account explanatory (i.e. mechanistic) evidence of *how* the drug produced this benefit. Notably, Bradford Hill did not take the positive results of his first RCT as establishing the effectiveness of streptomycin in this context precisely because the effectiveness claim was undermined by evidence of the mechanisms of antimicrobial resistance [95, 96].

Bradford Hill’s nine criteria for demonstrating causality comprise:

- strength of association (a strong association is more likely to be causal than a weak one);
- consistency (multiple measurements made by different investigators on different occasions in different contexts suggest a causal relationship);
- specificity (an outcome that is predicted by just one primary factor suggests that that factor causes the outcome);
- temporality (a cause must precede an effect);
- biological gradient (a ‘dose response’ relationship between the putative cause and the outcome suggests causality);
- plausibility (a relationship is more likely to be causal if there is a rational and theoretical explanation for it);
- coherence (an association is more likely to be causal if it coheres with other knowledge about the variables being studied);
- experimental manipulation (if the outcome changes when variables are changed in an experiment, causality is more likely); and
- analogy (a commonly accepted phenomenon in one field of study can sometimes be applied to another field) [96].

Mechanistic evidence contributes to many of these criteria, and particularly to the criterion of plausibility [12].

In sum, a robust demonstration of causality typically requires considering *both* associative (ideally, RCT) evidence *and* mechanistic evidence. While this idea is far from new, there has been little work undertaken prior to the work on Evidential Pluralism (and, derivatively, EBM+) on how to bring these kinds of evidence together beyond simple narrative synthesis. We hope that SR+ will contribute to filling this gap.

### Orthodox evidence synthesis: hierarchies and GRADE

In the ‘Background’ section above, we introduced the hierarchy of evidence beloved of the EBM community as an element of the *orthodox* approach to evidence synthesis. To our knowledge, the first version of this hierarchy was first published in 1998 (in the context of encouraging doctors to give more weight to RCTs than non-RCT evidence when assessing efficacy of drugs) [97]. It has subsequently been refined many times and formalised into various guides to good practice.

Notably, in 2004, the Grading of Recommendations Assessment, Development and Evaluation (GRADE) working group analysed and combined six previous evidence ranking systems to produce the GRADE criteria for assessing the trustworthiness of evidence when synthesising evidence (for example, when developing clinical guidelines) [98]. Over the next few years, a series of more than 30 papers published in the Journal of Clinical Epidemiology provided detail on how to assess and quantify the ‘risk of bias’ in different kinds of study (see for example [99–102]). The series also included papers on how to convert the results of its highly technical grading procedures into policy recommendations [103, 104] and communicate them to non-epidemiologists [105].

GRADE checklists quickly became the default method used by medical journals for benchmarking evidence synthesis submissions [1]. More than 70 organisations around the world, including the UK’s National Institute for Health and Clinical Excellence [106], the World Health Organisation [107], the US Agency for Healthcare Research and Quality (AHRQ) [108] and the international Cochrane Collaboration [109], have adopted the GRADE method to rank and assign various numerical risk-of-bias scores to each item of evidence considered. A new series of ‘core GRADE’ papers is currently being published in the BMJ [110–113].

While the GRADE system is now hard-wired into policymaking and guideline development, it focuses almost exclusively on associative evidence, recommending the use of mechanistic evidence only indirectly and warning reviewers not to place too much trust in such evidence. For example:

*‘The GRADE system does not rate evidence either up or down based on the mechanism or pathophysiological basis of a treatment. RCTs typically begin with a reasonable expectation of success based, to some degree, on biological rationale. But judgments of exactly how strong is the rationale are easily open to dispute, and GRADE does not suggest using them directly as a basis for rating evidence quality up or down. Mechanism does, however, have multiple roles in the evaluation of evidence: in selecting studies for systematic reviews, in the applicability of evidence to different interventions or populations, in judging whether to believe subgroup analyses, and in deciding the extent to which one rates down quality of evidence based on surrogate outcomes.’* (page 1309) [114]

This firmly articulated position by orthodox evidence synthesis authors overlooks the crucial contribution that mechanistic studies make to deciding whether an intervention works. Evidence of the presence of features of the purported mechanism can raise confidence that an observed association is in fact causal [3, 12]. Conversely, absence of these features, or presence of key features of counteracting mechanisms, can undermine confidence in effectiveness. Mechanistic evidence is also vital for optimising the design of interventions of various kinds, from drug therapies matched to the molecular structure of a biological target [17] to policies that comply with legal norms [115].

The architects of the GRADE system have been vocal in criticising other scholars and policy bodies of misappropriating the GRADE kite mark or ignoring or misapplying its principles and tools [116, 117]. In particular, they have expressed concern that ‘strong’ policy recommendations are frequently made on the basis of ‘weak’ evidence (which they define as lacking an estimate of effect size, or limited to an estimate from a single study with wide confidence intervals) [117]. While they concede that such ‘discordant’ recommendations are sometimes justified, they believe that such situations should be extremely rare—and they flagged in 2016 that they had classified 55% of all WHO guideline recommendations to be ‘discordant’.

Yet the GRADE authors also acknowledge that, in addition to the main defining feature of ‘strong’ evidence (confidence in an estimate of treatment effect), other factors may reasonably influence the strength of a policy recommendation. These could include, for example, ‘magnitude of the desirable and undesirable consequences of alternative courses of action, value and preference judgments required in trading off desirable and undesirable consequences, […] resource use considerations’ (page 99) and also ‘burden of illness, accessibility, feasibility, acceptability, barriers and facilitators for implementation, the extent of current suboptimal practice, and the impact on health inequities’ (page 99) [117].

GRADE offers a quantitative and objective way of considering resource use (by comparing published economic evaluations conducted in different settings) [118] and advocates the use of PROM and HRQOL metrics to capture (a version of) patients’ perspectives [119, 120], but nowhere in the GRADE guidance is there any formal advice on *how* the various contextual and ‘soft’ considerations alluded to in the aforementioned quotes might be brought into a local decision-making process.

The orthodox approach to guideline development (GRADE) thus appears open to enhancement by the systematic inclusion of mechanistic evidence. This would include academic knowledge from other disciplines (e.g. laboratory sciences, anthropology, human geography, policy studies, law) as well as the experience of practitioners, patients and citizens (e.g. through local consultation and deliberation exercises), all of which could generate mechanistic insights. We contend that a *systematic* approach to evaluating evidence of mechanisms, such as the SR+ approach to be tested in this study, might improve the consistency with which we assess such things as the implementation challenges of a multi-component intervention, community opposition to a particular policy or the role of legal mandates in achieving compliance with an intervention.

### Heterodox evidence synthesis: narrative and realist reviews

The orthodox approach to evidence synthesis described in the previous section is unambiguously monistic: it is rooted in clinical epidemiology, embraces quality standards oriented to the elimination of statistical bias and favours RCTs. In this section, we briefly summarise two other (‘heterodox’) approaches to evidence synthesis—narrative review and realist review. These are both explicitly pluralistic, embracing multiple forms of evidence and combining these in various ways, usually without using a formal evidence ranking system.

A narrative review is ‘a scholarly summary along with interpretation and critique’ (page 2) [121]. Whereas systematic reviews undertaken according to GRADE criteria are oriented to narrow (‘focused’) questions and designed for the purpose of *extracting and aggregating data* (especially, data on effect sizes), narrative reviews often address *broad* questions and are undertaken for a different purpose—*furthering understanding*. The GRADE-driven orthodox systematic review places limited emphasis on interpretation and critique—activities that may be particularly important when the review is intended to inform policies that are contested and even resisted. In such circumstances, a narrative review which surfaces and examines the multiple aspects of the topic, including how different interest groups frame the issues, may be as important as—and sometimes, more important than—a so-called systematic review.

Ogilvie et al. have used the metaphor of the ‘dry stone wall’ approach to depict how narrative synthesis can neatly draw together different ‘shapes’ and ‘sizes’ of evidence from different sources to provide a rich and unique overview of a topic (as opposed to a Cochrane review which seeks to build a ‘brick wall’ by aggregating as many similar studies as possible) [122]. Various specific methodologies for narrative review have been proposed, including hermeneutic reviews (such as qualitative comparative analysis [123], framework-based thematic synthesis [124], critical interpretive synthesis [125] and meta-ethnography [126]) which focus on creating a deep understanding of a topic in context; mixed-studies reviews (narrative reviews which combine qualitative and quantitative evidence) [127]; and meta-narrative reviews (over-arching reviews which explore how research on a topic has unfolded in different traditions over time, with early ideas and discoveries influencing subsequent work) [128, 129].

Narrative reviews have been criticised for lacking a systematic and reproducible methodology, potentially leading to recommendations that are not transparent and to conflicting reviews on the same topic (when two sets of authors interpret evidence differently). Search strategies and judgements (e.g. about which evidence to include or exclude, or how to weigh the importance of different evidence sources) can be made transparent to some extent, but since the essence of a narrative review is interpretation, a certain amount of variation in conclusions (and hence recommendations) is to be expected. The Cochrane Methods Handbook includes a chapter on ‘qualitative evidence synthesis’ focused mainly on how to synthesise qualitative evidence in process evaluations of RCTs (a form of mixed-methods review) [130]. These authors seek to align with orthodox systematic review methodology—for example, they propose a ‘risk to rigour’ tool analogous to the quantitative ‘risk of bias’ tool; they replace the PICO (population-intervention-comparison-outcome) structuring tool with PerSPECTiF (perspective-setting-phenomenon of interest-environment-comparison-time-findings); and they suggest using logic models to systematise the synthesis process. Such an approach, steeped in the language of epidemiology, has not been widely embraced by the qualitative research community.

Realist review is a form of systematic review which emphatically rejects the idea of a fixed effect size for complex social interventions such as policies [86, 131]. The realist reviewer asks ‘what works for whom in what circumstances?’ (in other words, for whom and in what circumstances will the effect size of this intervention be large, and for whom and in what circumstances will it be small, non-existent or even negative?). Rather than taking a linear view of causality (*X* → *Y* with a fixed and knowable effect size), the realist reviewer views causality as *generative* (i.e. assumes that particular outcomes emerge in a complex system when and to the extent that key contributing influences line up, but not otherwise). Realists are primarily interested in surfacing the mechanism (by which is usually meant the human interpretation and reasoning) through which a particular intervention or programme achieves its effect when circumstances are favourable. Realist analysis involves the use of abductive reasoning (asking questions like ‘what might explain…?’) and the examination of disconfirming cases to explore why the putative mechanism(s) failed to ‘kick in’.

Realist reviews are presented as designed to inform policymaking [132, 133] and the idea of realist review is popular with healthcare policymakers, but a realist review is labour-intensive (hence, expensive and time-consuming) to conduct and its main output may be an academic paper that is strong on methodological purity and abstract analysis but weak on practical implications [134, 135]. A ‘rapid realist review’ methodology has been developed [136] and some authors have begun to suggest ways for realist review to become more practically focused [134].

Both narrative review and realist review (in different ways) seek to develop and test theory; hence, they are sometimes categorised as ‘theory-based reviews’, which raises the question of what we mean by theory. The word can mean different things in different kinds of evidence synthesis. These include, for example, ‘grand theory’ (expressed at a high level of abstraction), ‘middle-range theory’ (designed to combine particular concepts in an empirical setting), ‘theory of change’ (i.e. programme logic, or how the designers of a programme think their intervention will work) or ‘realist theory’ (hypotheses about what works for whom in what circumstances, expressed as a set of context-mechanism-outcome configurations).

A novel approach to evidence synthesis, the argument framework, has recently been proposed by philosophers [137]. This approach, grounded in the philosophy of reasoning, seeks to strengthen narrative review by the use of formal rules of argumentation, with each step in the argument broken down into its constituent premisses, sub-premisses and sub-arguments, each of which will need to be tied to evidence. Gaps in the evidence base can be precisely identified by locating the sub-premises and sub-arguments for which sufficient data are lacking. This technique has only recently been described (somewhat coincidentally, its authors used masking as a brief example) and there are no published worked examples of its application.

One contribution of the study described here will be to delineate the strengths and weaknesses of SR+ vis a vis narrative review, realist review, other forms of theory-based review and the argument framework.

### Epistemic injustice

The term ‘epistemic injustice’ was proposed by Fricker to denote situations in which someone is wronged in their capacity as knower [8]. She originally depicted two kinds of epistemic injustice: *testimonial injustice*, that is, attributing too little credibility to a speaker on the basis of prejudice through negative stereotyping (e.g. when someone who is old, female, Black, gay, ill or disabled is assumed to have limited knowledge as a result of these characteristics); and *hermeneutic injustice*, when a speaker lacks the conceptual resources to make sense of and communicate their experience (e.g. when a member of a marginalised group is unable to step forward and articulate their experiences in a way that more mainstream actors find persuasive). Testimonial injustice, to the extent that it can be traced back to prejudice, is a form of direct discrimination; hermeneutic injustice is an example of indirect discrimination [8]. The literature on epistemic injustice has been criticised for overlooking *social* injustice [138] and downplaying racial and colonial injustices [139]. In many cases, one group’s epistemic justice is won at the expense of another group’s epistemic *injustice*. More recently, Fricker has suggested extending the concept of epistemic injustice to include a third category, *distributive injustice*, where disadvantaged people have less access than others to goods, such as education or expert advice, which would help them convey their evidential claims [140].

Evidence-based medicine’s hierarchy of evidence, which infamously either omits or downgrades qualitative studies, real-world studies and the experiential evidence of patients (especially disadvantaged and marginalised ones) [141], has been critically examined using an epistemic justice lens [5–7]. Broadly speaking, the argument goes that so-called evidence-based (objective, controlled, reproducible, linked to a body of established body of formal knowledge) criteria for diagnosing, investigating and managing medical conditions or risk states systematically downgrades the phenomenological and *subjective* evidence of people placed in these categories.

The downgrading or dismissal of qualitative research studies (however well-designed and well-conducted) in orthodox systematic review undoubtedly raises an *epistemic* objection, since qualitative research can provide important mechanistic evidence (e.g. about the filtration properties of mask materials or the reasons for non-compliance with masking). Whether this downgrading or dismissal counts as *epistemic* injustice is less clear. Fricker’s definition of epistemic injustice is sometimes narrowly interpreted to apply only to the downgrading of patients’ and lay people’s evidence as offered directly (e.g. when patients sit on guideline panels), but Fricker herself allowed that prejudice against another researcher’s method can constitute epistemic injustice [8].

In sum, we anticipate that this study will contribute to the debate on whether orthodox review is *ethically* questionable by virtue of epistemic injustice in additional to being epistemically questionable.

### Outputs and dissemination

We anticipate that the study will generate multiple academic papers and conference presentations, both empirical (relating to the face masks example) and methodological (relating to evidence synthesis and the philosophy of causality). We plan to produce explicit guidance and publication standards for SR+ and negotiate their dissemination via with the EQUATOR (Enhancing the QUAlity and Transparency Of health Research) network, a central repository of methodological guidance for all kinds of empirical and secondary research relevant to health.

In terms of non-academic outputs, we plan to engage directly with the policy and regulation community. Because SR+ has the potential to inform policy, we will take steps to ensure that key agencies are aware of SR+ (including its applications, strengths and limitations compared to other review methods). From the outset, we will build relationships with key policy and advisory bodies whose remit includes developing, implementing and evaluating complex interventions, and we will ensure that there is ongoing dialogue with such bodies. Bodies include the UK Government Evaluation Task Force, the UK Evaluation Society, UK What Works Centres, Parliamentary Select Committees for Post-Legislative Scrutiny and the European Commission’s Evidence-Informed Policy Making team. Other non-UK bodies will be invited to join our workshops and engagement events online.

We will also engage with providers and advocacy groups. We anticipate that SR+ will provide tools for those who advocate for the disadvantaged and underserved, for whom the evidence base may be heterogeneous and contested. For example, the question of who should mask under what circumstances plays out differently for clinically vulnerable groups (e.g. cancer patients), who are more susceptible to catching an infectious disease and also more likely to die or incur serious harm [142]; health and care workers, who are at occupational risk of infectious diseases and include a high proportion of minority ethnic groups and low-paid workers [143, 144]; and people with long COVID, for whom reinfection with SARS-CoV-2 may produce flare-up of their condition [145]. All these groups have sought to present evidence (of various kinds) to support the argument that masking should not be left entirely to the personal choice of healthy and low-risk individuals. Such groups, and those who advocate on their behalf, need accessible tools to support their arguments that the absence of definitive RCT evidence should not be used to stall policies that are likely to benefit them. We will proactively identify groups who may benefit from SR+ and engage them in dialogue.

A major component of our dissemination strategy is providing training in the methods of SR+. Once the SR+ methodology has been developed, we will produce a range of training materials and organise three one-day workshops, one for a general academic audience, one for evaluators and policymakers, and one for advocacy groups.

### Strengths and limitations

A key strength of this study is its interdisciplinary basis and its grounding in sound philosophical principles. The core research team includes experts in analytic philosophy (and, especially, the philosophy of Evidential Pluralism), public health (especially, the science of masking), orthodox systematic review (Cochrane review), narrative review, realist review, law (especially, the emerging sub-discipline of evidence-based law [146]) and epistemic justice (especially the contribution of patient-based evidence and local and indigenous knowledge to deliberation on policy issues). Another strength is that the study is well placed to fill an important evidence gap, in that SR+ may be especially helpful in crisis policy situations and other times when rapid decisions are needed but RCT evidence is lacking or non-definitive.

However, while interdisciplinary working is a theoretical strength, it raises operational challenges. Scholars from different disciplines make different assumptions about the nature of reality, about what counts as ‘quality’ research and about how evidence should be identified and synthesised. This can lead to misunderstandings and conflicts. We will emphasise dialogue and reflexivity among our team and will seek to capture any tensions that emerge among us as data.

Another strength of this study is its potential to provide nuance on evidence-based policies. For policy questions, there is rarely a single and universal answer based on ‘science’ (e.g. an effect size that can be determined through an RCT). Rather, policy contexts are typically complex and require interpretation and application of science, including attention to what is known about mechanism. Key to policy decisions is external validity (generalisability)—the extent to which findings from a RCT will apply in contexts beyond those under which the trial was conducted. To answer that question requires a range of evidence, especially evidence that captures the complexities of context. We hope that SR+ will help make consideration of this wider evidence base more systematic.

One potential limitation of our study is that findings from a single in-depth example (face masks) may not be universally generalisable to other evidence synthesis challenges. However, we selected this example as a ‘critical case study’, defined by Yin as one which, for whatever reason, will be decisive in testing a theory (i.e. if the theory ‘works’ in that case, it is likely to work in most or all cases) [147]. Face masks are a critical case study here because (a) there is a large amount of evidence, from multiple different study designs; (b) there is much controversy about how to rank and synthesise that evidence; (c) the question of who should mask and in what circumstances, and when (if at all) masking should be legally mandated will be of the utmost public health importance in the next major infectious disease outbreak; and (d) there are clear ethical issues, with certain vulnerable and marginalised groups standing to be disadvantaged if their evidence continues to be overlooked. We acknowledge, however, that the methodology of SR+ will need to be further tested on additional complex case studies, and are currently exploring candidate examples which would provide complementary philosophical challenges.

## Conclusion

As the controversy over mask evidence has illustrated, the current orthodox system of evidence review (using Cochrane/GRADE methodology) has a number of potential shortcomings, including concerns about external validity and the injustice of rejecting or downgrading qualitative studies and service user experiences. We hope that our proposed alternative, Systematic Review+, will provide a systematic and reproducible way of integrating associative and mechanistic evidence drawn from across a number of disciplines and study types. In this way, we hope to provide a way of achieving a more accurate understanding of the effectiveness of interventions and the means by which they achieve their effect (or not).

## Supplementary Information

Supplementary Material 1.

## Data Availability

The review will be based on published studies which are therefore accessible to all. Details of any specialised analyses will be provided to bona fide researchers on reasonable request to the corresponding author.

## Appendix

### Details of search strategy and data management

#### Search strategy

Searching will be iterative and use multiple methods. These will include:

- keyword search of at least 8 databases (Medline, Cinahl, Cochrane, Psychinfo, SSCI, SCOPUS [148], JSTOR, Annals of Work Exposure and Health);
- author search (authors of seminal papers will be name-searched in relevant databases to identify additional papers by them);
- citation-tracking (via Google Scholar);
- mining previous systematic reviews;
- relevant engineering standards and the references on which they are based, including British/European (e.g. EN14683, EN149), American (e.g. National Institute for Occupational Safety and Health, NIOSH) and Canadian (e.g. Canadian Standards Association, CSA);
- asking experts in relevant fields, e.g. occupational health and safety.

Keywords and database search strings:

Related to masks: masks, respirators, face coverings, non-pharmaceutical interventions (NPIs), respiratory protective devices.

Related to respiratory outbreaks: respiratory outbreak, respiratory pandemic, epidemic control, public health intervention,

Specific respiratory illness keywords: COVID-19, SARS-CoV-2, influenza, SARS (Severe Acute Respiratory Syndrome), MERS (Middle East Respiratory Syndrome).

Outcome and effect keywords: transmission rate, infection rate, disease spread, hospitalization rate, mortality rate, community transmission, effectiveness, impact, efficacy.

Mechanism keywords: aerosols, air filtration, filtration efficiency, adsorption, absorption, breathability, fit factor, mask efficiency, mask coatings, antibacterial.

Database search strategies:

Combine keywords using Boolean operators (AND, OR, NOT).

Example: (mask*) AND (COVID-19 OR influenza) AND transmission rate

Use truncation (*) to capture variations of words.

Example: mask* will find masks, masking, etc.

Use phrase searching (quotation marks) to find exact phrases.

Example: public health intervention

Use MeSH terms (Medical Subject Headings) in PubMed/MEDLINE for more precise results.

Example search strings:

PubMed: (mask*[Title/Abstract] OR face covering[Title/Abstract]) AND (COVID-19[MeSH Terms] OR influenza[MeSH Terms]) AND (transmission[Title/Abstract] OR infection[Title/Abstract])

Google Scholar: mask* AND (respiratory outbreak OR pandemic) AND (transmission OR effectiveness)

These keywords and search strategies will be piloted in the specific databases and modified in response to emerging findings.

#### Data management

Data will be stored on University of Manchester, University of Oxford and University of Exeter computers. Eligible papers will be stored, organised and coded on an Endnote database. Data extraction will occur using summaries on Microsoft Office packages (e.g. Word, Excel). Specialist data management packages compliant with university data policies will be used if needed.
